# Preceptorship and Affirmation in the Intergenerational World of Nursing Practice

**DOI:** 10.1155/2012/572510

**Published:** 2012-06-13

**Authors:** Vicki Foley, Florence Myrick, Olive Yonge

**Affiliations:** ^1^School of Nursing, University of Prince Edward Island, 550 University Avenue, Charlottetown, PE, Canada C1A 4P3; ^2^Faculty of Nursing, University of Alberta, Edmonton, AB, Canada T6G 1C9

## Abstract

Research has shown that while preceptorship offers a reality-oriented learning environment and facilitates competence of students, there are inherent rewards and stressors associated with the experience. Students and preceptors can be from different generations, and as such, they may often come to the learning space with differing values and expectations. The nature of the preceptorship experience in this intergenerational context was explored in a recent phenomenological study with seven preceptors and seven nursing students in an undergraduate nursing program in Eastern Canada. Overall the experience was found to be inclusive of three main themes: *being affirmed, being challenged*, and *being on a pedagogical journey*. In this paper we explore the first of these themes, *being affirmed*. Highlighting the positive aspects of the preceptorship experience in the intergenerational context is necessary to promote a culture of openness and respect for generational differences within clinical nursing practice settings and to improving the overall quality of the educational experience.

## 1. Introduction

Research has shown that while preceptorship offers a reality-oriented learning environment and facilitates competence of students, there are inherent rewards and stressors associated with the experience [1]. The formation of a positive working relationship between a student and a preceptor highly influences the overall success of the preceptorship experience. In today's nursing clinical practice settings, there can be up to four generations (Silents, Baby Boomers, Gen Xers, and Millennials) present and each can be said to have its own distinct worldview [2]. The majority of today's university and college students belong to the Millennial Generation (also referred to in the literature as Generation Y), while most preceptors are either Baby Boomers or Generation Xers. Within the discipline of nursing, values and expectations are often deeply rooted in traditions and customs of nursing practice and invariably as the younger generation brings new ideas to the practice setting, clashes between the generations are occurring and these can be difficult to resolve [3]. Promoting a positive community of practice for nursing students is the shared responsibility of nurse educators and preceptors [1, 4]. 

In this phenomenological study, the experiences of seven nursing students and seven preceptors within the intergenerational world of nursing practice were explored. Participants were recruited from an undergraduate nursing program in Eastern Canada. The findings revealed that the overall experience was inclusive of three main themes: *being affirmed, being challenged, *and* being on a pedagogical journey.* The purpose of this paper is to undertake an in-depth exploration of the first theme, *being affirmed. *A total of five subthemes emerged from the data in relation to this theme. From the students' perspective, being affirmed related to *havinga professional role model*and *building confidence. *The preceptors' reflections related more specifically to *being respected, imparting the legacy, *and* strengthening nursing knowledge. *These five subthemes will be explored in more detail and supported with direct quotes from the participants that are believed to be particularly illuminating. In our view, highlighting the positive aspects of the preceptorship experience in the intergenerational context is necessary in order to promote a culture of openness and respect for generational differences within clinical nursing practice settings. Knowledge of the affirming aspects is also directly relevant for recruitment and retention of preceptors, as well as student satisfaction with the clinical experience. Such knowledge can assist nurse educators as well as they prepare both students and preceptors for the preceptorship roles and responsibilities.

## 2. Background

At best, limited research on the intergenerational context of the teaching and learning process in higher education has been conducted and, in particular, no published studies have been carried out on preceptorship or field education in the intergenerational context [5]. A lack of research is also noted in the area of preceptorship rewards, specifically in relation to working with someone of a different generation. There is some literature available which more broadly identifies the rewards of preceptorship from the view of the preceptor [4, 6–14]. These studies reveal both intrinsic and extrinsic rewards of being a preceptor. Some examples of intrinsic rewards include seeing student growth and development, opportunities to strengthen teaching skills and influence practice, improve own knowledge base and critical thinking skills, and the opportunity to reflect on and evaluate one's own practice. Extrinsic rewards identified include pay differentials, opportunities for continuing education, journal subions, luncheons, and other similar initiatives. It is suggested in the literature that rewards must be individualized and those that are deemed to be personally/professionally meaningful contribute most to continued commitment to the preceptor role [15, 16]. 

The affirming nature of preceptorship from the point of view of the preceptee has not been examined specifically; however in one phenomenological study, the lived experience of student nurses in relation to learning was explored, and three main themes were identified: *directing learning, learning in practical action*, and *feeling in learning* [17]. These themes provide unique insight into the different modes of learning from the student's perspective. While no rewards were mentioned per se, the authors highlight the important responsibility of nursing faculty in providing “real life” positive situations for students. One other study (not specific to preceptorship) examined the experiences of nursing students in relation to professionalism [18]. A total of 69 baccalaureate nursing students at different educational levels participated in the study and the researchers reported that “knowing” and “belonging” were affirming for students in their professional role. Overall, a review of the literature reveals that there is limited research available on the topic of preceptorship and affirmation. Owing to this, it is difficult to ascertain whether any of the affirming aspects identified in the literature relate directly to being precepted by/precepting someone from a different generation. Findings from the current study help to fill this gap in the literature.

## 3. Research Methodology

Phenomenology was the methodology employed for this study. The goal in phenomenological human science research is to develop a rich, deep interpretation of lived experience that enables externalization of that which is internal and may not have been reflected upon previously [19]. Phenomenological researchers strive to make such insight available to others who have a similar interest in the phenomenon. This study was guided by van Manen's [19] approach to phenomenology. van Manen draws from the works of influential phenomenological philosophers such as Husserl, Heidegger, Gadamer, Merleau-Ponty, and others, to offer methodological guidance for researchers interested in conducting phenomenological inquiry. His particular approach is more “action sensitive” than philosophical and he promotes conducting human science research to specifically inform and improve pedagogy. van Manen notes that phenomenological reflection presents the possibility of thoughtful praxis through individual and collective self-understanding. 

## 4. Research Questions

Two main phenomenological questions guided this research study (1) What is it like to precept a student who is of a different generation? (2) What is it like to be precepted by a nurse who is of a different generation? 

### 4.1. Sample

To recruit participants for this study, the principal investigator worked in collaboration with the Clinical Placement Coordinator (CPC) of an undergraduate nursing program in Eastern Canada where the principal investigator was formerly employed. The principal investigator was the coordinator of the third year preceptorship course for a period of about six years and was familiar with many of the preceptors and students. The principal investigator and CPC met to purposely identify participants who would be open and willing to discuss their experiences. The goal in purposive sampling is to deliberately look for “information rich cases that capture analytically important variations in the target phenomenon” and select participants based on their knowledge and ability to communicate about their experience of the phenomenon under investigation ([20, page 181]). With this in mind, we chose students of different age ranges (i.e. Millennials and Generation X), and preceptors of different age ranges (i.e., Generation X and Baby Boomers) as well as both males and females. In order to avoid any perceived coercion, the invitations to participate in the study were distributed by the CPC via email. 

Small sample sizes (e.g., 6–10) are characteristic of phenomenological studies [21] as the goal is not to make external generalizations about the population, but to attempt to gain deep insight into the meaning of the lived experience [19, 22]. In this study, there were 14 participants: seven of these were nursing students and seven were preceptors. Of the seven students, four were female, three were male, two were Gen Xers, and five were Millennials. Of the seven preceptors, six were female, one was male, and all were Gen Xers. All of the preceptors had at least five years of experience precepting students and the student participants were all in their final year of their nursing program and were reflecting upon their preceptorship experiences in both the third and fourth years of the program. It was our goal to recruit participants from both generations of preceptors; however none of the Baby Boomers responded to the invitation. It is important to acknowledge as well that at the time of data collection, the third year preceptorship course had been completed and the principal investigator was no longer working directly with any of the participants and was not responsible for the student evaluations. None of the preceptors who participated in this study had worked directly with any of the student participants. Both groups were informed at the outset that we would not be interviewing any participant that they had been paired with previously. To further maintain confidentiality and to ensure anonymity, pseudonyms were assigned to each participant.

### 4.2. Data Collection

Data were collected during two unstructured interviews with each of the participants. The interviews served the dual purpose of collecting data to discover a rich, deep understanding of the preceptorship experience in the intergenerational context, as well as creating a dialogue between the researcher and the participants about the meaning of their experience. Following the first round of interviews, a number of preliminary themes became apparent and, during the second interviews, these themes were further explored in order to confirm and extend the analysis. Participants were also guided to reflect upon the meaning of their experience. Ethical permission to conduct the study was granted by the university research ethics board.

### 4.3. Data Analysis

van Manen [19] describes his approach to human science research as being derived from both the German tradition of human science pedagogy (the Dilthey-Nohl School), which employed an interpretive methodology, as well as the Dutch movement of phenomenological pedagogy (the Utrecht School), which was noted to be more of a descriptive methodology. In order to distinguish his particular approach to human science research, van Manen identifies an active and ongoing interplay of six distinct research activities: (1) turning to the nature of the phenomenon, (2) investigating the experience as we live it rather than as we conceptualize it, (3) reflecting on the essential themes which characterize the phenomenon, (4) describing the phenomenon through the art of writing and rewriting, (5) maintaining a strong and oriented relation to the phenomenon, and (6) balancing the research context by considering parts and whole. 

As researchers, we engaged in each of these six activities and moved back and forth between them as the project unfolded. Data collection and analysis occurred simultaneously. We became immersed in the data by listening to tapes and reading/rereading the transcripts. Following the first round of interviews the research team met to discuss the emerging themes and to flesh out what gaps existed that might need to be explored during the second interviews. We prepared a summary of the emerging themes and included some direct quotations from participants that were found to be particularly revealing. This summary was sent to all participants and we then requested a second interview to explore these further. Following the second interviews, we revised the thematic analysis and began to structure the phenomenological text. We drew from van Manen's [19] suggestions when deciding how to structure our research text. He identifies five possible approaches: (1) thematically, (2) analytically, (3) exemplificatively, (4) exegetically, and (5) existentially. He adds that these are not mutually exclusive or exhaustive and that researchers may choose a combination of approaches or may invent an alternative organization. 

We chose to structure our phenomenological text using a combination of the thematic and analytical approaches as outlined by van Manen [19]. In the thematic approach, the emerging themes serve as generative guides for writing the research report and the totality of the research findings are explicated under each thematic heading with the recognition that there is inherent overlap among the themes. Complex phenomena are further explored using subsuming themes as was the case in our study. The analytical approach may take several forms; however for our purposes we considered etymological sources of the key words in each of the three main themes to further explicate the meaning of the theme. 

We acknowledge that juxtaposing human experience with categories or themes can be challenging in that one must be careful not to attempt to “fit” all experiences into the identified categories. Throughout the study we were mindful of the need to keep a fresh perspective on the experiences of preceptors and students through a process of constantly revisiting the tenets of phenomenology within the context of the preceptorship experience that was occurring. It was important for us to acknowledge that choosing an epistemological lens by the way of themes or categories was merely a means to organize the text and we recognize that not all experiences would necessarily fit neatly under each of the categories. The challenge for us was to treat each theme systematically while keeping in mind that one theme always has meaning dimensions in other themes. van Manen also cautions that deciding which themes are essential versus incidental is one of the greatest challenges in structuring the text. In so doing, we were ever mindful of the need to ensure that the main themes fit with each person's experience. 

### 4.4. Rigor and Phenomenological Research

Methodological rigor is an ongoing issue in qualitative research and many authors suggest that it is inadequate to apply a quantitative concept of rigor to that of qualitative research [19, 23–28]. van Manen [19] posits that the criteria for rigor and rationality in human science research cannot be the same as that of natural science research because a much broader view of rationality is essential. Subsequently, he proposes four evaluative criteria which seem most appropriate for judging the power and convincing validity of any phenomenological human science texts. He states, “Our texts need to be oriented, strong, rich, and deep” ([19, page 151]). 

In this study, we aimed for the strongest possible interpretation of the phenomenon through our awareness of the human lived experience and through the writing of a text deep in meaning. We believe that the data analysis will enable the externalization of our participants' lived experience. Reflexivity on the part of the researchers was instrumental in maintaining a strong and oriented relation to the phenomenon. Reflexivity relates to self-awareness and the acknowledgement that the actions and decisions of the researcher invariably impact upon the meaning and context of the phenomenon under investigation and is also a means of showing honesty and transparency in the research process [27, 29]. During this study, we reflectively explored our observations, prereflections, taken for granted assumptions, critical thinking, and decision-making. In particular, we acknowledge that we have made assumptions at times that the different generations do not understand each other. In our view, a lack of awareness of generational differences can lead to tension and/or conflict in the preceptor-student relationship. Such tension can have a negative impact on student learning as well as the overall success of preceptorship. It is also our view that promoting understanding of generational diversity is an important responsibility of nursing faculty. 

Husserl [30] used the term “bracketing” to describe how researchers must put aside any preexisting knowledge or assumptions they may have about the phenomenon; however van Manen [19] questions whether it is realistic for researchers to truly put aside their knowledge of the subject. He posits that it is not necessary to “bracket” the information, but rather researchers have a responsibility to make their knowledge of the phenomenon explicit. He goes further to suggest that presuppositions may resurface into the researcher's reflections when we try to forget that which we already know. Throughout this study, we sought to make our personal knowledge of the phenomenon more explicit through the use of a reflective journal. 

 To further ensure rigor, our participants were afforded the opportunity to confirm and extend the initial interpretations during the second interviews. Our participants not only openly shared their experiences, but also engaged in an interpretive conversation/relation with the researchers and, during the second interviews, we believe a “phenomenological nod” was evident. Munhall [31] refers to the “phenomenological nod” as participants nodding in agreement when reading or listening to the study findings, which is indicative that their experience has been captured by the researcher (page 189). One other means of ensuring rigor was to examine the study themes in the context of current nursing research literature [22] and, in so doing, we found our themes to be supported by the work of other researchers. 

## 5. Findings and Discussion 

In this study, the phenomenon of preceptorship within the intergenerational world of nursing practice was found to be inclusive of three main themes: *being affirmed, being challenged*, and *being on a pedagogical journey*. As mentioned previously, our purpose in this paper is to examine the first of these themes in depth. To begin, let us explore the meaning of the phrase *being affirmed*. From an etymological point of view, the word “affirm” derives from the French *afermer* and from the Latin *affirmare, *meaning to make steady, strengthen, and consolidate [32]. More specifically, the term “affirm” is defined as “to validate, confirm, state positively with confidence, declare as a fact, assert to be true” ([33, page 35]). In the context of this study, *being affirmed* relates directly to the participants' experiences of being validated, strengthened, and consolidated, all of which were identified as rewards of either precepting or being precepted by someone from a different generation. 

After asking participants to describe their own generation and what they like about that generation, we also explored their thoughts about other generations. Inherent in the reflections of all participants was the notion that the overall experience was an *affirming* one. As one student remarked:

*It was fantastic. I've had really good experiences… never once did I feel from them [preceptors] that they put me down or made me feel any less than what I was—which is still a student… I could always go to them with any kind of question, there was never a stupid question. So they were really open-minded and flexible when it came to that. I always ask questions… I like to seek out my own experiences, but they're always the same, looking for experiences for me to take part in… I just found it was great, I didn't have any complaints.” (Christina) *

Similarly, another student commented:

*I didn't have any encounters that were bad at all. They [preceptors and staff] were really accepting, and they were happy that I was interested and eager for learning and wherever they could help out, they helped out… a really positive experience. (Ashley)*

Another student, Kayla, also described her preceptorship in the intergenerational context as a very positive experience and felt that her preceptor's up to date knowledge base and positive attitude toward the younger generation was refreshing. She stated:

*She was very welcoming to students. She didn't have that negative attitude that ‘oh students are lazy' because I think she had really good experiences in the past. She even told me ‘every student I've had in the past was really good and worked hard' so I kind of think that that worked to my benefit. She was still really up to date too…so I found that was good too...she was really knowledgeable. (Kayla)*

One of the Gen X students was afforded the unique opportunity of working with a preceptor of the same generation. He reflected on his experience as follows:

*My first preceptor for the 3rd year course was actually my own generation—X, which was great, because we kind of had the same attitudes, I feel anyway. I think it went great… I loved the whole experience. (Mark)*

Preceptors also highlighted the *affirming* nature of their experience with precepting younger students. Patricia stated that it was “absolutely positive overall…I feel young, I love it, and I've gotten nothing but respect from them.” Karen agreed and identified that her experience was:

*Definitely positive overall…the students that I have had, for the most part, have been quite eager to learn and, you know, they're very intimidated initially, but once you make them feel comfortable and you teach them the best you can, you know, for the most part, I haven't had any issues.*

It is important to note that *being affirmed *was manifested differently for the students than the preceptors and there were six subthemes identified which illuminate this affirmation more clearly. From the students' perspective, being affirmed related to *havinga professional role model*and *building confidence.*

### 5.1. Having a Professional Role Model

Nursing students often look to the clinical practice setting or the “nursing world” for affirmation of their professionalism ([18, page 81]). In this study, students explained that they believed that they worked very well with experienced nurses and appreciated the practical wisdom and knowledge, as well as the sense of pride in the nursing profession that older nurses embodied. Andrew's comment suggests that students want to emulate the professional approach of experienced nurses.

*I wanted to go to somebody older than me. I wanted someone who has a sense of the profession, and a model of professionalism which is maybe a bit more old-fashioned model perhaps than some of the younger generation. I've learned skills and knowledge from all nurses, but that issue of finding someone to model professionalism…for me, that's the part that I've appreciated the older generation the most for.*

Similarly, Mark stated:

*Actually it was pretty rewarding because, I felt that I could learn a lot from the older generation. They have the skills built up from many generations, really. And she [preceptor] was willing to…share whatever she had, so it was great that way. I had a really positive experience overall.*

Mark added that knowing he could rely on his preceptor led to feelings of comfort and security which was *affirming* for him in his student role. He further reflected on his experience as follows:

*I had somebody to go to if I got in trouble…not in trouble, I should say, there was no trouble, but if I had questions, I had that contact there, and she was more than willing to give me information and help me through whatever I had issues with. *

Christina also recognized that older nurses have a wealth of nursing knowledge and experience and, as such, represent significant role models for students. She did suggest that personality, as well as age or generation, can play a role in determining whether the preceptorship experience would be a positive one. She elaborated in this way:

*Well, it depends on their own personality, of course. But the ones I've lucked into having, I've just been in total awe of them, to be quite honest. I just find that they're really knowledgeable and confident in their work and everything about their work, really. And the ones I've had have been really open minded to giving me as much exposure, or as much freedom, I guess, to explore my own type of experiences. *

### 5.2. Building Confidence

For the students in this study*, being affirmed* was also manifested in being afforded the opportunity to build their confidence. Preceptorship in the intergenerational context was seen as a direct embodied experience or, in other words, a planned immediate encounter in the here and now, that involved them physically and emotionally [34]. This direct embodied experience allowed them to build confidence in their ability to perform new roles. Students' reflections on their experiences as noted in this subtheme are consistent with the constructivist view of experiential learning, particularly Kolb's [35] cycle of learning, which involves four phases: concrete experience, reflective observation, abstract conceptualization, and active experimentation. The first two phases, concrete experience and abstract conceptualization, are considered to be “two dialectically related modes of grasping experience,” while the other two phases, active experimentation and reflective observation, are viewed as “two dialectically related modes of transforming experience” ([36, pages 193-194]). During the learning cycle, immediate or concrete experiences serve as the basis for observations and reflections which are then integrated and refined into abstract concepts from which new implications for action can be drawn. These implications can then be actively tested thus serving as guides in creating new experiences and building on previous learning [36]. 

Kolb et al. [36] note that conflict between the “concrete” or “abstract” and between “active” or “reflective” is generally resolved in patterned ways based upon individual learning styles. Kolb [35] identifies a learning style inventory which can be used to assess four distinct learning styles known as diverging, assimilating, converging, and accommodating. We posit that all four phases of Kolb's [35] cycle of learning were evident in the students' comments. For example, Justin described how comfortable he *felt* with his Gen X preceptor's level of guidance.

*There was never a time when she was watching me that I was uncomfortable, or that when I went on my own I was uncomfortable. (concrete experience)*

Sarah commented on the importance of *doing* and stated, “That's one of the main benefits I see…just getting out there and being able to put your skills in action…that's something that I appreciated” (active experimentation). She added that her preceptor's level of experience working with students was a real strength and it was a key factor in allowing her to progress more easily.

*My preceptor in third year—she's been preceptoring for ten years—so she's very experienced: a) in what the outcomes of the course are, so she was very good at helping me meet the objectives of the course, because of that was very aware of my scope of practice—what I could and couldn't do—those types of things. She was also very comfortable with students, and I think that's a factor as well. If you have a nurse that's never preceptored before, it's essentially a person doing work under your umbrella, under your name, but it's not you doing it. And I could see that nurses would be very uncomfortable with that–I would be uncomfortable with that. So if you're not used to having a student, that can be an uncomfortable thing.*

Similarly, Andrew concurred, “You do sort of learn a huge amount in your first real clinical placement with nurses and that has been really valuable for me” (reflective observation). Andrew added, “The preceptorship experience…was the beginning of feeling like a nurse” (abstract conceptualization). 

Kayla identified her preceptor's strength in relation to giving feedback and recognized this as a significant contribution to developing her own confidence. She also reflected upon experiences with other nurses that were not so positive.

*She [preceptor] was great, because in other rotations I've had other nurses who said “I'm not one to praise someone up but if you're doing something wrong I'll tell you”. But she was good because if I was uncertain about a skill or it was my first time doing it I would kind of verbalize to her first, if I was nervous or really unsure, she would come in and watch me or if she thought…actually, no …she's just watch me and afterwards say “okay, you did that really good” or “make sure next time that you got the bed up higher so you're not bending over”, stuff like that.*

Another student noted that her preceptor's openness and receptiveness went a long way toward building her confidence.

*My preceptor last year, she was 45, and I found that she was excellent…if I said something, she was open to it, and I was so open to everything she said, so I thought we kind of got along really good and we could learn from one another…and I felt that I got a lot more independence and I thought that…when I finished, I felt like I was ready to be a nurse. (Ashley) *

Overall, the students' recognition of their growth toward increasing confidence led to a feeling of *being affirmed*. This affirmation as it relates to the presence of a professional role model as well as building confidence, as found in this study, is congruent with previous research [10, 18, 37–41]. Kolb's [35] learning cycle is reported to correspond well with field education experiences in professional disciplines [42] and in nursing in particular, research on experiential learning theory dates as far back as the late 1970s [43]. 

All seven of the preceptors in this study also agreed that precepting younger students is an *affirming* experience. In particular, they described the following aspects of the experience to be most significant and these are also considered to be subthemes of *being affirmed*: *being respected, imparting the legacy, *and* strengthening nursing knowledge.*

### 5.3. Being Respected

With regard to feeling respected for their knowledge and skill, one of the preceptors indicated that he felt valued when students and new nurses choose to come to him when they have questions about nursing care. He stated:

*They come to me, and I've always had that, after I got so many years under my belt, lots of people come to me, and still do, and I like that because I'd rather for them to come to me and ask, and if I don't know, I'll get the answer rather than them go make a mistake. So I encourage that. (Dave)*

Another preceptor noted that it is “very rewarding to see a student who's just left the area and they're so impressed with the experience that they had and they thank you for passing on some of your knowledge” (Lisa). Such “tokens of gratitude” are considered to be a significant reward of precepting ([7, page 6]). 

### 5.4. Imparting the Legacy

Being able to impart the legacy of nursing onto the younger generation was another significant reward for preceptors and led to feeling *affirmed*. One preceptor noted, “I like teaching those that are coming up, the things that I already know, that I wish I knew when I began” (Colleen). She added:

*And I like learning the new stuff that they come to me with because there's always new stuff in nursing. They learn it and I learn it from them. “Oh we learned how to do it this way”, “that's a good way, I'll remember that.”*

Another preceptor elaborated on the personal responsibility she perceived for imparting the legacy and noted that she felt satisfaction when students achieved success. She stated:

*It kind of reflects back on me when a student is finished and going to soon graduate and become a nurse and whatever type of nurse they are if they spent 8 weeks with me, obviously I've had some impact on them somehow in what they do…and how they behave…and when you find out that you know what, this is a great person, we're so happy we hired them, it's almost like a satisfaction for yourself. (Wendy)*

Likewise, Lisa stated, “hopefully you will go with them wherever they go. Like, they'll look back at it [the preceptorship experience] and say, you know, I learned a lot from this preceptor.” 

Students in this study also acknowledged the preceptors' innate ability to impart the legacy of nursing onto them. For example, Andrew observed, “I've seen it…they [preceptors] care about nursing…and they want to impart something onto the next generation of nurses.” This genuine commitment to the profession and demonstrating value for the education of future nursing professionals could be said to exemplify what Myrick et al. [39] refer to as “engaging in authentic nursing practice,” a process which the authors suggest nurtures practical wisdom in the preceptorship experience (page 82). These authors note that affirming the student role and recognizing student potential were inherent in the preceptor student interaction and were key elements of engaging in authentic practice.

### 5.5. Strengthening Nursing Knowledge

Several of the preceptors highlighted the benefit of feeling as though their own nursing knowledge was strengthened as a result of working with nursing students. Wendy remarked, “It keeps me on my toes and it keeps me up on my skills and reminds me of why I'm in there too. It makes me appreciate what I do.” Lisa described students as “a wealth of knowledge when they're coming out of school.” She added, “I am impressed with them.” Colleen indicated that she appreciates it when students inform her of new ways of doing things. Patricia specified that learning these new things and keeping current made her “feel so good!” She went on to say:

*I find that I am so on top of all my policies. I just find that I'm on top of everything, I'm so educated and I learn alongside my preceptee. They bring you new knowledge and new ideas. So you're more open, so it's good both ways, you know? You can learn.*

The preceptors described many other positive attributes of working with students of the Millennial Generation. Dave commented on the fact that he looks forward to “an influx of new blood” each time a new group of preceptees comes to his nursing unit and this keeps him from feeling “stagnant.” He elaborated in this way:

*I really have no problems working with the younger graduates. I mean, I look forward to it, it's an influx of new blood almost, because sometimes you get stagnant in areas and then the new people come in, and sometimes when they come in they're right ready to take charge and raring to go. Sometimes you need that little jolt in units … I have no problems, none whatsoever, I like new people coming in. Just change. Change is good for any area, you know.*

Sharon expressed that she enjoys working with students because “they're fun to be around and they are high energy, and I like being challenged.” She added:

*I like working with younger people because even at my age, I'm 46 years old, but at my age I don't consider myself old. Now I know a 20 year old looks at me and thinks “oh grandma” (laugher), but I don't consider myself old. I like to think that as long as your mind is active and your mind is progressive and you're open to new ideas, you're not old. So I enjoy working with the younger people because they're fun. They're fun to be around and they are high energy, and I like being challenged. I like to be in a situation where I like to know your stuff, because you've got to pass it on to someone else and you've got to teach it to somebody else. And they ARE very inquisitive.*

Lisa added, “They keep you young, keep you vibrant, keep you educated and…they're the ones we're going to be depending on.” 

Overall, these comments reveal a level of self-reflection that emanates from being a preceptor and such reflection in and of itself was *affirming* for preceptors. Preceptors recognized that they were strengthening their own knowledge base and awareness of themselves as professional nurses. This finding is corroborated by other researchers who have specifically examined the experiences of preceptors [9, 14, 44, 45]. Numerous other intrinsic rewards such as teaching, role modelling, contributing to the future of the profession, moulding students, and enhancing one's pedagogical skill through precepting are also identified [7, 8, 12, 13, 46–49]. 

## 6. Implications and Recommendations

Findings of this study generate new knowledge about the different generations, namely, Millennials and Gen Xers, and the influence of generational diversity in shaping the teaching/learning process in the clinical practice setting. The three themes identified serve to provide the structure and meaning of the participants' lived experience. The first theme, *being affirmed*, showed that both preceptors and students found the experience to be positive and rewarding and highlighted the fact that the rewards were manifested differently for each group. Identifying the affirming aspects is significant as it allows others to understand the nature of the rewards inherent in the experience and further research in this area would be beneficial to strengthening pedagogical nursing knowledge. It is important to acknowledge that a number of *challenges* were identified in this study as well; however it is outside the scope of this paper to address the challenges that were revealed. We acknowledge as well that developing strategies to address the challenges is crucial to the future success of the preceptorship model of clinical practice. As well, further research addressing the specific challenges that relate to preceptorship in an intergenerational context is warranted. 

Overall, we believe this study provides a beginning understanding of the different generations and how they work together in preceptorship, but there is clearly a need for further research in this area. One of the topics that we suggest pursuing is a broader assessment of generational perceptions within the nursing profession. A descriptive exploratory study with a large sample of preceptors and students across multiple nursing schools within Canada would be highly valuable in adding to this important body of nursing knowledge. We also advocate conducting other phenomenological studies to examine experiences of different dyads, such as Baby Boomers preceptors and Millennial students, or Millennial preceptors and Millennial students. Such research would help to elucidate whether the challenges identified in the current study are indeed related to the intergenerational context of the learning space. The impact of generational diversity on workplace leaning is another important area to be explored. Interprofessional studies to examine experiences of field educators and students from other disciplines would allow for valuable comparisons to be made with the findings of the current study. It is important to raise the question of whether the challenges described in this study are unique to nursing thus interprofessional studies would aid in establishing a foundation for knowledge utilization. 

## 7. Limitations

This study describes the lived experiences of seven nursing students and seven preceptors as they negotiate the teaching/learning process in the intergenerational context. The complexities of their collective experiences have been illuminated, but that is not to say that another phenomenological study with a different group of preceptors and students would yield the same findings. The preceptors in our study were all of the Generation X and all had at least 5 years of experience with precepting students. It is likely that their experiences differ from those of less experienced preceptors and perhaps preceptors of other generations, such as Baby Boomers. 

We also acknowledge the inherent limitation of any human science research that a description of one's experience, even a rich and deep description, can never truly capture the entirety of that lived experience. van Manen [19] admonishes that we must “remain aware that lived life is always more complex than any explication of meaning can reveal” (page 18). It is also worthy of mention here that we are not suggesting that generational diversity is the most important issue in nursing preceptorship today, but rather it is an emerging topic of interest and one that we believe warrants further attention. It is difficult to ascertain whether the rewards and *affirming *aspects of the preceptorship experience as described above relate specifically to working with someone of a different generation or rather preceptorship from a broader perspective. We should also add that many challenges were identified by our study participants and we recognize that a generational lens is only one way to view situations where conflicts and/or challenges are occurring. Such situations can be highly complex and the generation to which a person belongs is merely one factor in the equation. We did not explore personality conflicts or differences in learning styles in this study per se; however these topics were raised by some of our participants.

We recognize one other limitation, the difficulty in reaching all fourteen of the participants for a second interview. Following the first round of interviews a written summary of emerging themes, along with some direct quotations from the interviews, was sent to each participant and a second interview was requested to confirm, extend, or challenge the analysis. We were successful in receiving feedback from all but two of the participants (one preceptor and one student). 

## 8. Conclusion

This study lays the foundation for pedagogical nursing knowledge development in the area of generational diversity. Through the phenomenological methodology as described by van Manen [19], we have sought to provide a rich, deep interpretation of the real-life experiences of both preceptors and students as they negotiate the teaching and learning process in the intergenerational world of nursing practice. Three essential themes were identified (*being affirmed, being challenged, *and* being on a pedagogical journey*), each consisting of a number of subthemes. Our purpose in this paper was to explore the theme, *being affirmed*, in a rich and deep way, and we believe that the findings and analysis we have presented here allow for externalization of our participants' experiences. Each of the participants in our study described their lived experience as an *affirming* experience and one that reaped many personal rewards. We believe that highlighting the *affirming* aspects of this experience is significant for the sustainability of the preceptorship model of clinical education. Generational diversity undoubtedly influences the teaching/learning process in preceptorship and developing further understanding of this phenomenon is directly relevant for nurse educators, students, and nurses in clinical practice. The findings of our study can be used to improve the preparation of students and preceptors for the intergenerational world of nursing practice. As Raines [50] notes, “We can use generational lenses to help us see things we might not otherwise notice” (page 2). 
